# Trace Li Pre‐Doping of Activated Carbon Cathode Enabling Accelerated Li‐Ion Transport for High‐Power Lithium‐Ion Capacitors

**DOI:** 10.1002/advs.76990

**Published:** 2026-08-10

**Authors:** Kyungmin Do, Jihyeon Ryu, Dong Gyun Im, Seong‐Yong Kim, Tae‐Hyun Kwon, Byeong Guk Kim, Seojin Jeon, Woosun Jang, Myoung Joon Hwang, Ki‐Hun Nam, Seung Yol Jeong, Ick‐Jun Kim, Seungwook Eom, Jaeik Hyun, Sunhye Yang

**Affiliations:** ^1^ Battery Research Division Korea Electrotechnology Research Institute (KERI) Changwon Republic of Korea; ^2^ Electric Energy Materials Engineering KERI School University of Science and Technology (UST) Daejeon Republic of Korea; ^3^ School of Chemical Engineering Pusan National University Busan Republic of Korea; ^4^ Department of Battery Engineering Yonsei University Seoul Republic of Korea; ^5^ Integrated Science & Engineering Division Yonsei University Incheon Republic of Korea; ^6^ H/C R&D Division VINATECH Jeonju Republic of Korea; ^7^ Nano Hybrid Technology Research Center Korea Electrotechnology Research Institute (KERI) Changwon Republic of Korea

**Keywords:** activated carbon cathodes, high‐power energy storage, interfacial lithium transport, lithium pre‐doping, lithium‐ion capacitors

## Abstract

Rapid expansion of AI‐driven data centers demands energy storage systems that simultaneously deliver high power, safety, and practical scalability. Lithium‐ion capacitors (LICs) are attractive candidates; however, their performance is fundamentally constrained by inefficient Li pre‐doping and pore blockage in activated carbon (AC) cathodes. In this study, ultralow‐level Li pre‐doping, far below conventional loading thresholds, is demonstrated to induce substantial performance enhancement without compromising the intrinsic porous structure of AC. Through a simple Li‐based surface modification followed by controlled thermal conversion, an ultrathin and uniformly distributed lithiophilic layer is introduced, where Li_2_CO_3_ is identified as the most effective phase for high‐power operation. Remarkably, even trace Li incorporation, undetectable by conventional spectroscopic techniques, significantly enhances Li‐ion transport, as supported by molecular dynamics simulations. This minimal yet effective surface modification reduces polarization while improving both capacity and rate capability. Consequently, pouch‐type full cells exhibit enhanced high‐power performance, increased capacity, and stable cycling behavior under practical operating conditions. These findings establish Li pre‐doping as a scalable and cost‐effective strategy for engineering high‐performance LIC cathodes and provide a viable pathway toward next‐generation energy storage systems for AI‐driven infrastructure.

## Graphical abstract

Trace‐level Li pre‐doping on activated carbon cathodes is demonstrated to significantly enhance Li‐ion transport and high‐rate performance in lithium‐ion capacitors. An ultrathin Li_2_CO_3_ interfacial layer, formed through controlled thermal conversion, facilitates rapid ion transport without pore blockage. The optimized electrodes deliver outstanding rate capability and long‐term cycling stability in practical pouch‐type full cells.

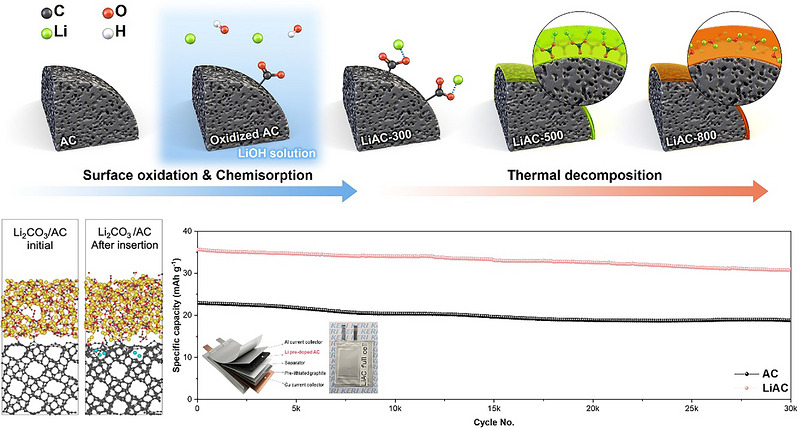

## 1 Introduction

The rapid growth of AI‐driven computation is accelerating the construction of large‐scale data centers, resulting in a substantially increasing demand for high‐power energy storage systems which ensure stable and continuous power supply under highly dynamic operating conditions [1, 2]. Lithium‐ion batteries (LIBs) have received significant attention as dominant energy storage systems owing to their high energy density and long cycle life. However, their relatively low power capability and inherent safety concerns remain critical challenges, particularly for applications requiring high charge–discharge rate and robust operational conditions [3, 4, 5]. In contrast, electric double‐layer capacitors (EDLCs) offer exceptionally high‐power density and outstanding safety but suffer from intrinsically low energy density, which limits their applicability to large‐scale energy storage [6, 7, 8, 9, 10]. Lithium‐ion capacitors (LICs) have emerged as promising hybrid energy storage devices which combine the LIBs and EDLCs, exhibiting high energy density of batteries with the high power and safety of capacitors [11, 12, 13, 14, 15].

Typically, LICs employ activated carbon (AC) as the cathode due to its high surface area and excellent capacitive behavior, while various materials such as graphite, hard carbon (HC), soft carbon (SC), and metal oxides have been explored as anode candidates. In LIC systems, the AC cathode stores charge through the adsorption of anions, whereas lithium ions must be supplied from the anode side during the initial cycles to enable reversible operation and to realize the full capacity of the device. Therefore, a Li pre‐doping process for the anode is considered an essential step in LIC fabrication [16, 17, 18]. Several approaches have been reported to achieve Li pre‐doping, including the use of porous metallic current collectors that allow lithium ions to penetrate through the pore structure and pre‐dope the electrode, as well as electrochemical lithiation of the anode prior to cell assembly. However, these approaches face practical limitations for commercialization due to the high cost of porous metallic current collectors and the low efficiency associated with electrochemical pre‐doping processes.

To overcome the remaining limitations of LICs, recent studies have increasingly focused on Li pre‐doping strategies to improve Li‐ion kinetics and energy‐storage capability. In typical LICs, the charge–discharge process is governed by asymmetric ion‐storage mechanisms between the battery‐type anode and the activated carbon (AC) cathode. At cathode potentials above the potential of zero charge (PZC), charge is mainly stored through the adsorption of electrolyte anions within the ion‐accessible porous structure of AC [19]. As the cathode potential decreases during discharge, these adsorbed anions are released, and below the PZC, Li^+^ ions migrate from the anode to the AC surface and contribute to additional charge storage. Upon subsequent charging, the adsorbed Li^+^ ions move back to the anode, restoring the original adsorption state of the AC cathode. This dual ion‐storage behavior allows LICs to combine high power characteristics with improved energy‐storage capability. Accordingly, promoting Li^+^ storage on the cathode side has emerged as an effective approach to simultaneously enhance both the power and capacity of LICs [19, 20].

Li pre‐doping of the AC cathode has emerged as a promising strategy to mitigate the intrinsic limitations of LIC systems. In this context, various Li‐containing compounds have been introduced into the AC cathode as sacrificial lithium sources for pre‐lithiation, such as Li_2_MoO_3_, Li_5_FeO_4_, Li_3_N, and Li_2_C_4_O_4_ [13, 17, 21, 22]. Beyond this additive‐based approach, surface engineering of activated carbon has also been actively explored to regulate electrode/electrolyte interfacial reactions and improve the electrochemical stability of LIC cathodes. In particular, Li‐containing interfacial species, including Li‐ion‐conductive compounds such as LiAlO_2_, can construct lithium‐friendly interfacial domains that facilitate Li‐ion transport and stabilize interfacial behavior. Various approaches, including liquid‐phase mixing and solid‐state reactions, have been employed to introduce these Li‐containing species onto AC surfaces [23, 24, 25]. Such surface‐modified AC cathodes have been shown to effectively reduce polarization, improve rate capability, and enhance cycling stability, highlighting the potential of lithiophilic surface engineering for high‐performance LICs. However, this strategy is highly dependent on the type and distribution of Li compounds on the AC surface. Because AC possesses a porous structure and a large specific surface area, excessive loading or non‐uniform deposition of Li compounds can block ion‐accessible pores and deteriorate the intrinsic high‐power characteristics of AC [15, 26, 27, 28]. Therefore, uniform and ultrathin deposition of Li compounds is essential to preserve pore accessibility while introducing lithiophilic functionalities. From a practical perspective, the amount of Li precursor must also be minimized to ensure process scalability, cost‐effectiveness, and environmental compatibility.

In this work, we present a cost‐effective strategy to enhance the capacity and power density of LIC cathodes under practical operating conditions through an ultralow‐level Li pre‐doping of AC. The LiOH precursor was employed to introduce Li‐containing functionalities on the surface of AC, which were subsequently converted into Li compounds via thermal decomposition. By tuning the heat‐treatment temperature, the chemical state of the resulting Li compounds was systematically controlled, and Li_2_CO_3_ formation was found to deliver the most favorable power properties. Notably, even an undetectable small amount of Li pre‐doping led to a pronounced improvement in electrochemical performance, allowing us to identify a minimal coating condition that is highly compatible with practical and scalable processing. Molecular dynamics simulations further revealed that such undetectable Li incorporation can significantly promote Li‐ion transport within the AC framework. Finally, pouch‐type full cells assembled with the optimized Li pre‐doped AC cathode and a pre‐lithiated graphite anode demonstrated high power capability, improved capacity, and stable cycling performance under practical conditions. These results highlight the strong potential of the proposed approach for next‐generation high‐power energy storage systems, particularly for emerging applications such as AI data centers that demand both high power density and reliable long‐term operation.

## 2 Results and Discussion

### 2.1 Synthesis of LiAC and Phase Evolution of Li Compounds

**FIGURE 1:**
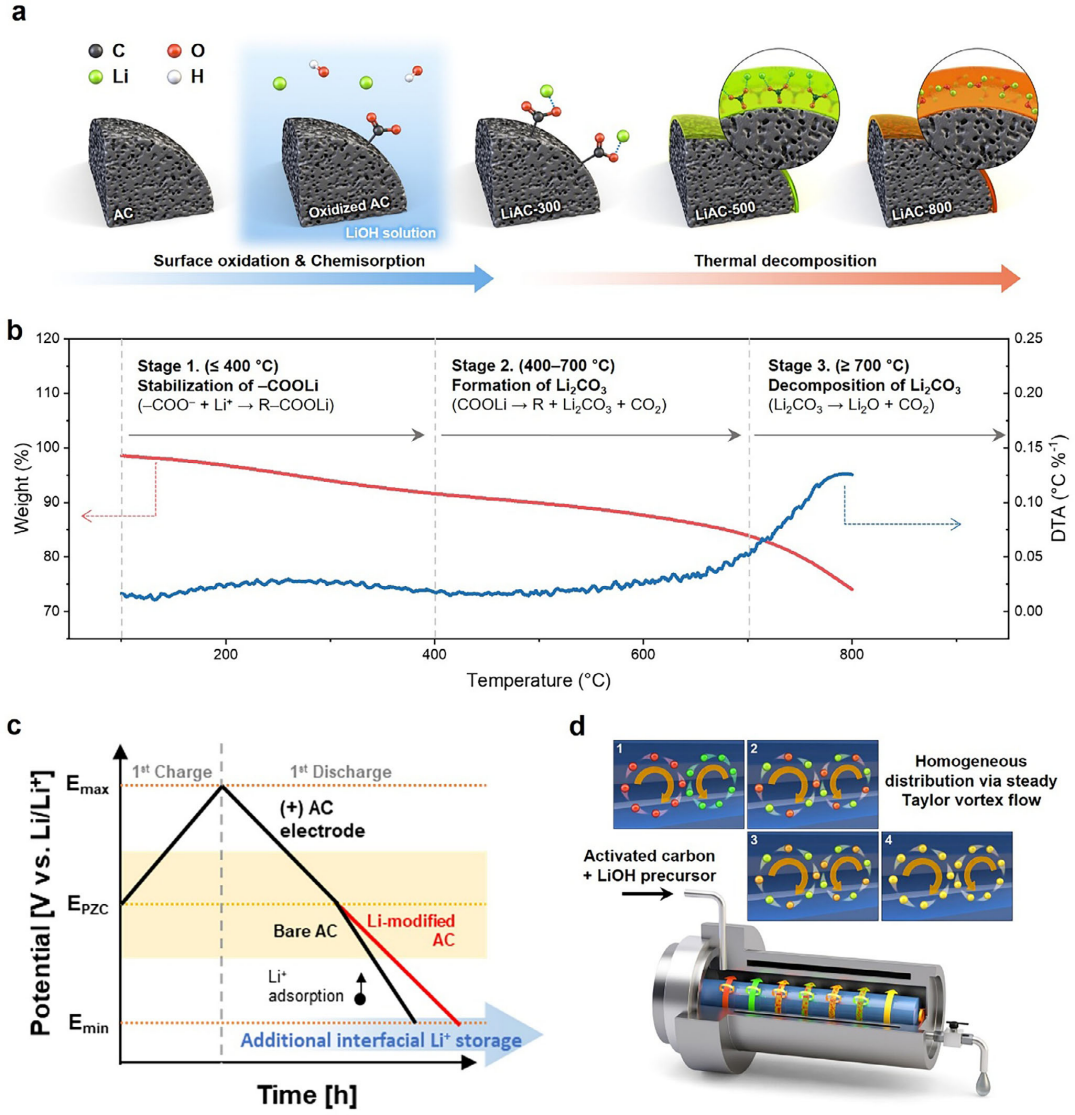
Preparation of LiACs. (a) Schematic illustration for Li pre‐doping and thermal decomposition process of Li‐compounds. (b) TGA and DTA curves of LiAC measured under Ar atmosphere with the temperature range from 100°C to 800°C. Thermal decomposition proceeds in three distinct stages: (1) stable RCOOLi below 400°C, (2) formation of Li_2_CO_3_ at 400°C–700°C, and decomposition to Li_2_O above 700°C. (c) Schematic illustration of the evolution of voltage profiles with Li pre‐doping. (d) Couette‐Taylor reactor for homogeneous mixing.

Figure 1a schematically illustrates the synthesis strategy for lithium‐doped AC (LiAC), in which Li compounds are formed on the surface of AC through the chemisorption of Li precursor followed by the thermal decomposition process. To promote effective adsorption of the Li cations, the surface of AC was mildly oxidized to introduce oxygen‐containing functional groups, particularly carboxylate (─COO^−^) species [29, 30, 31, 32]. This surface facilitates the adsorption of Li cations via electrostatic interaction and surface complexation, enabling uniform distribution of the Li precursor. After sufficient interaction, the Li‐loaded AC was subjected to thermal treatment at 300°C, 500°C, and 800°C to induce the formation of Li compounds on the surface of the AC, and the resulting samples were denoted as LiAC‐300, LiAC‐500, LiAC‐800, respectively. During this process, the adsorbed Li precursor undergoes temperature‐dependent thermal decomposition and transformation, leading to the formation of distinct Li‐compounds at different temperatures. The thermogravimetric analysis (TGA) profile reveals that the thermal decomposition process proceeds through three stages (Figure 1b). At temperatures below 400°C, R–COOLi species formed on the surface remain thermally stable without noticeable decomposition. As the temperature increases above 400°C, a decomposition reaction occurs, leading to the formation of Li_2_CO_3_. Upon further heating to temperatures above 700°C, Li_2_CO_3_ undergoes an additional decomposition process, resulting in the formation of Li_2_O [33, 34, 35, 36]. The morphological evolution associated with each stage is provided in Figure S1. For the LiAC‐300, the lithium precursor is uniformly adsorbed across the AC surface, resulting in conformal surface coverage that partially blocks the porous structure without the formation of distinct crystalline features. When the heat‐treatment temperature is increased to 500°C, the R–COOLi undergoes thermal decomposition, leading to the formation of particles distributed over the carbon surface, while the evolution of CO_2_ re‐exposes the previously blocked porous structure of the AC. Further increasing the temperature to 800°C does not lead to significant changes in the overall morphology; the particles remain well preserved on the surface. Furthermore, electron energy loss spectroscopy (EELS) elemental mapping confirms the uniform distribution of Li on the AC surface, indicating the homogeneous formation of Li‐containing compounds over the AC surface (Figure S2). Figure S3 presents the nitrogen adsorption–desorption isotherms of the pristine and LiACs. The pristine AC exhibits a typical type‐I isotherm with a high specific surface area, and LiAC‐300 exhibits a reduced specific surface area due to partial blockage of the microporous structure. In contrast, LiAC‐500 and LiAC‐800 undergo thermal decomposition of the Li precursor, which re‐exposes the porous structure and results in surface areas comparable to that of pristine AC.

The charge‐storage behavior of porous AC is dictated by ion electrosorption, where competitive adsorption–desorption of PF_6_^−^ anions and Li cations take place. As schematically illustrated in Figure 1c, a Li pre‐doping strategy is introduced to modulate the interfacial chemistry of AC and to enhance the charge‐storage process. During the first charge, the AC positive electrode is polarized above the point of zero charge (E_PZC_) toward the potential limit (E_max_), leading to the electrosorption of PF_6_^−^ anions within the porous structure. During discharge, the adsorbed PF_6_^−^ anions are released until the electrode potential reaches the PZC, after which Li cations are adsorbed until the reduction potential limit (E_min_) is approached. In this context, Li‐modified AC surface provide additional interfacial Li‐ion storage and facilitate Li‐ion transport, thereby increasing the capacity while simultaneously improving the rate capability of the electrode. To ensure uniform adsorption of Li ions across the surface, AC and the Li precursor solution were reacted in a Couette‐Taylor reactor [37, 38, 39]. The Couette–Taylor reactor is based on the flow generated between two concentric cylinders under rotational motion. Above a critical rotational speed, the flow transitions from laminar to well‐defined Taylor vortices, which induce enhanced radial and axial mixing. These vortex structures provide uniform shear and efficient mass transport, thereby enabling homogeneous distribution of reactants. In addition, its established use in continuous‐flow processing highlights its scalability.

**FIGURE 2:**
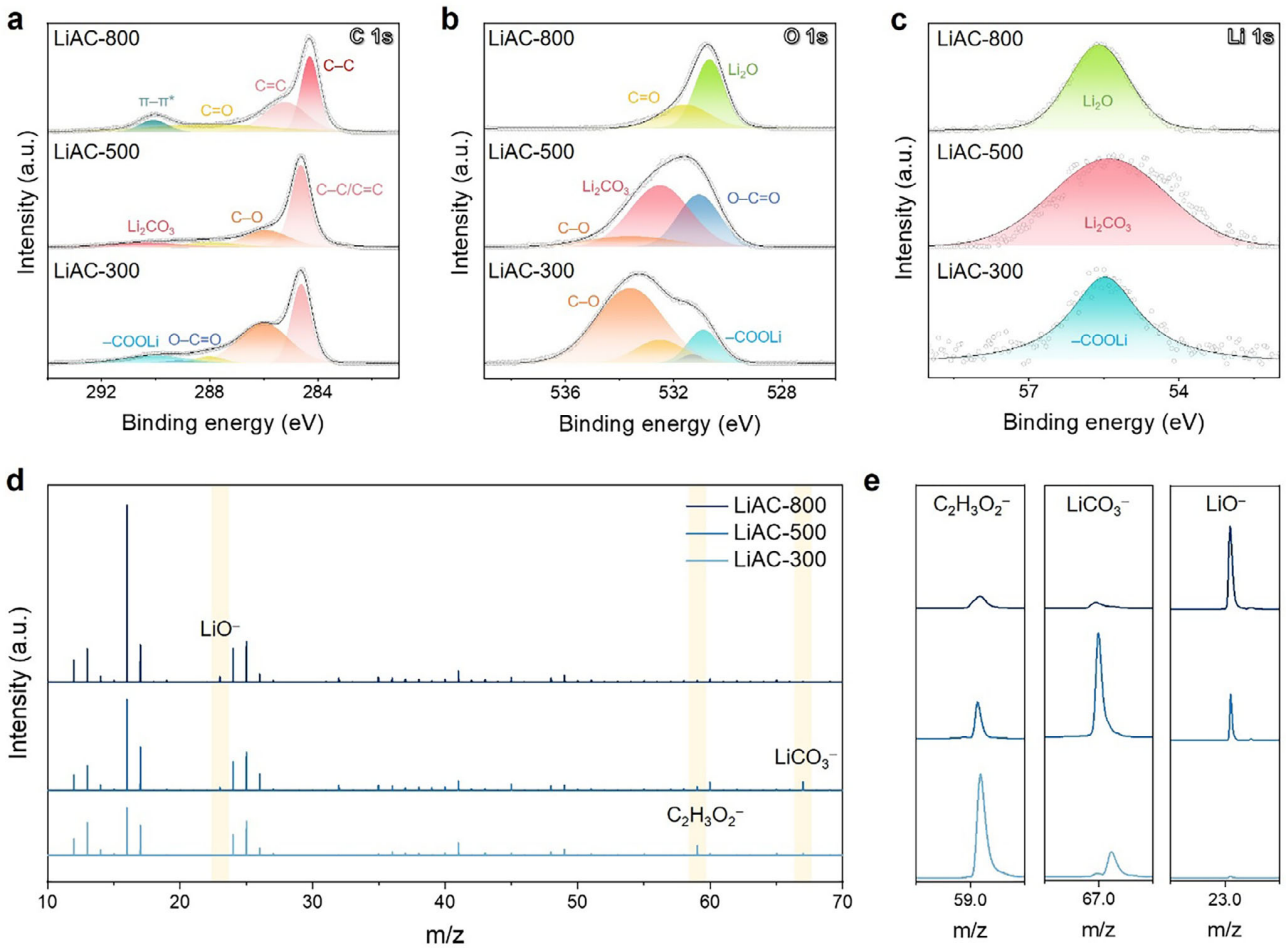
Thermally induced phase evolution and surface characterizations of LiACs. XPS spectra of (a) C 1s, (b) O 1s, and (c) Li 1s for LiAC‐300, LiAC‐500, and LiAC‐800. (d) ToF‐SIMs mass spectra of LiAC‐300, LiAC‐500, and LiAC‐800. (e) Corresponding enlarged regions of the mass spectra for C_2_H_3_O_2_^−^, LiCO_3_^−^, and LiO^−^ fragments.

The chemical state of the pristine AC, oxidized AC, and heat‐treated LiACs was analyzed by XPS (Figure 2a–c and Figure S4). The mild oxidation treatment induces a transformation of C═O bonding into C─O bonding, indicating the formation of a surface chemistry more favorable for Li adsorption. Upon subsequent Li doping on the oxidized AC, the C 1s spectrum obtained from LiAC‐300 shows three predominant peaks at 284.6, 286.0, and 290.0 eV, which are assigned to C─C, C─O, and ─COOLi bonding, respectively [40, 41]. As the heat‐treatment temperature increased to 500°C, a distinct peak at 290.3 eV emerged, which can be attributed to Li_2_CO_3_ formed via the thermal decomposition of –COOLi. Increasing the heat treatment temperature to 800°C, the residual –COOLi was completely decomposed, leading to the disappearance of the corresponding peak, implying the formation of other thermodynamically stable Li compounds at elevated temperatures. A weak π–π* shake‐up satellite is observed in the C 1s spectrum, suggesting the presence of aromatic sp2‐conjugated carbon domains [13, 42]. This feature is consistent with the graphitic character of the activated carbon framework. The O 1s spectra further reveal the evolution of Li compounds with increasing heat‐treatment temperature. The –COOLi peak observed from the LiAC‐300 disappears in LiAC‐500, accompanied by the emergence of a Li_2_CO_3_ peak, while further heat treatment at 800°C leads to the appearance of a Li_2_O peak. These results indicate the stepwise thermal decomposition of Li compounds, which is corroborated by the corresponding phase evolution observed in the Li 1s spectra.

To further investigate the phase transition on the surface, time‐of‐flight secondary ion mass spectrometry (ToF‐SIMS) depth profiling was performed on the LiACs (Figure 2d,e). The mass spectra exhibit distinct variations depending on the heat‐treatment temperature, with peaks at m/z values of 59, 67, and 23 assigned to C_2_H_3_O_2_^−^, LiCO_3_^−^, and LiO^−^, originating from –COOLi, Li_2_CO_3_, and Li_2_O, respectively [43, 44, 45, 46, 47]. For LiAC‐300, the C_2_H_3_O_2_^−^ peak is dominant, indicating that –COOLi is the primary Li species present on the surface. In contrast, LiAC‐500 exhibits the strongest LiCO_3_^−^ peak intensity, suggesting the predominant formation of Li_2_CO_3_ on the surface after heat treatment at 500°C. For LiAC‐800, the LiO^−^ signal becomes dominant, supporting the predominant formation of Li_2_O^−^ derived surface species after high‐temperature treatment. In addition, ToF‐SIMS mapping confirms that the dominant fragments at each temperature are homogeneously distributed over the surface (Figure S5). These results clearly demonstrate that surface LiCOO^−^ progressively decompose in the temperature range of 300°C–500°C to form Li_2_CO_3_, while a further phase transformation from Li_2_CO_3_ to Li_2_O occurs at temperatures above 500°C.

### 2.2 Controlled Trace‐Level Li Pre‐Doping of AC Cathodes

**FIGURE 3:**
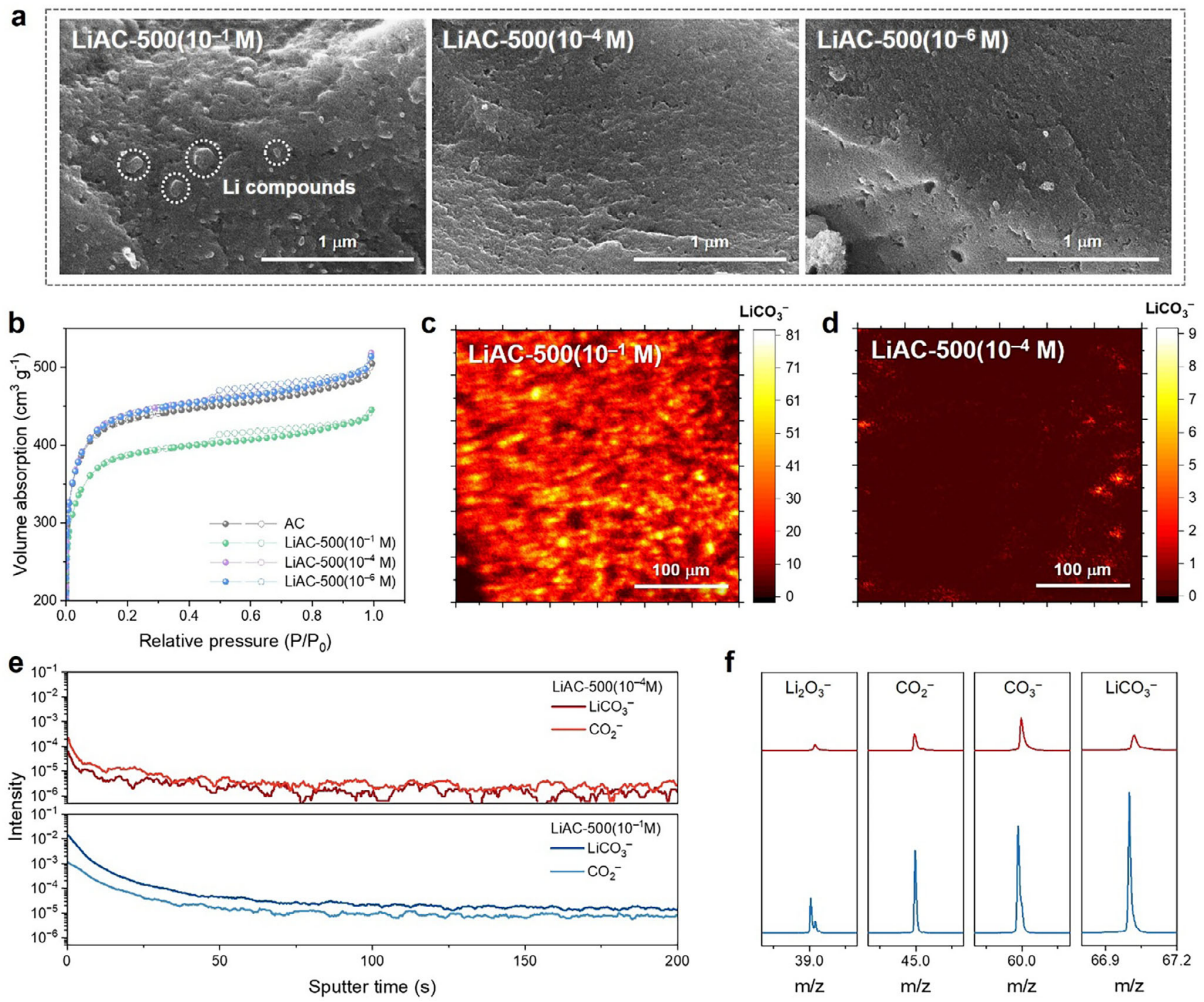
Effect of precursor concentration on the morphology and surface characterizations. (a) SEM images of LiAC‐500s prepared with precursor concentrations of 10^−1^ m, 10^−4^ m, and 10^−6^ m. (b) N_2_ adsorption–desorption isotherms of the corresponding samples. ToF‐SIMS chemical mapping showing the spatial distribution of LiCO_3_^−^ on the surface for the (c) LiAC‐500(10^−1^ M) and (d) LiAC‐500(10^−4^ m). (e) ToF‐SIMS depth profiles of LiAC‐500(10^−1^ m) and LiAC‐500(10^−4^ m). (f) Corresponding mass spectra highlighting Li_2_O_3_^−^, CO_2_^−^, CO_3_^−^, and LiCO_3_^−^ fragments.

The preceding results were obtained using a relatively high Li precursor concentration (1.0 × 10^−1^ m) to elucidate the adsorption behavior of Li and the subsequent evolution of Li compounds via thermal decomposition. Nevertheless, such high concentrations inevitably lead to excessive deposition of Li compounds, partially blocking the intrinsic porous structure of AC and degrading its electrochemical properties. To regulate the Li pre‐doping level, the concentration of the precursor was reduced to 1.0 × 10^−4^ m and 1.0 × 10^−6^ m during the Couette‐Taylor mixing process, while the thermal treatment temperature was fixed at 500°C to investigate the influence of Li_2_CO_3_ content. Accordingly, the samples thermally treated at 500°C using precursor concentrations of 1.0 × 10^−1^, 1.0 × 10^−4^, and 1.0 × 10^−6^ were denoted as LiAC‐500(10^−1^ m), LiAC‐500(10^−4^ m), and LiAC‐500(10^−6^ m), respectively. The surface morphology of LiAC‐500 prepared with different concentrations of Li precursor was investigated by SEM (Figure 3a and Figure S6). The LiAC‐500(10^−1^ m) exhibits a morphology in which the intrinsic pores of AC were partially covered and the Li‐compound particles formed on the surface, indicating that excessive Li precursor leads to the overloading beyond homogeneous surface coating. In contrast, when the precursor concentration was reduced to 1.0 × 10^−4^ m and 1.0 × 10^−6^ m, no distinct Li‐compound particles were observed while the surface morphology remained similar to that of pristine AC, suggesting that only an ultrathin coating layer was formed on the AC surface without altering the original porous structure.

The use of a relatively concentrated precursor solution results in the formation of a distinct surface layer on the AC, accompanied by the appearance of particulate Li‐containing compounds on top of the layer. These particles are indicative of excessive Li species, suggesting that an overly high precursor concentration leads to Li overloading beyond surface saturation. When the precursor concentration is reduced, no such particulate features associated with excess Li are observed. Instead, the AC surface appears to be uniformly covered by a thin and continuous layer, implying that Li species are well‐dispersed and preferentially anchored to the carbon surface without inducing secondary phase aggregation. In contrast, a further decrease in precursor concentration to an extremely dilute level results in an inhomogeneous surface morphology, indicating insufficient Li supply to achieve continuous surface coverage. These morphological observations are well corroborated by the BET analysis (Figure 3b). The LiAC‐500(10^−4^ m) and LiAC‐500(10^−6^ m) samples exhibit a typical type‐I adsorption–desorption isotherm, closely resembling that of pristine AC. This behavior suggests that Li‐derived species are deposited only as an ultrathin surface layer, thereby preserving the intrinsic microporous structure of the AC. By contrast, the LiAC‐500(10^−1^ m) shows a pronounced reduction in specific surface area, which can be attributed to pore blocking by excessive Li compounds accumulated on the AC surface.

At such low precursor concentrations, however, Li is incorporated at levels below the detection limit of conventional analytical techniques, including EELS and XPS (Figures S7 and S8). Nevertheless, ToF‐SIMS analysis revealed the presence of trace amounts of Li, while solid‐state ^7^Li nuclear magnetic resonance (NMR) spectroscopy further confirmed Li‐containing environments through a broad resonance near 0 ppm, indicating that the Li‐containing layer is extremely thin and present only in ultralow quantities on the surface (Figure S9). To investigate the distribution of Li‐compound formed under different concentrations of LiOH precursor, ToF‐SIMS analysis was conducted for LiAC‐500(10^−1^ m) and LiAC‐500(10^−4^ m). As shown in the ToF‐SIMS mapping images of LiCO_3_^−^ (Figure 3c,d and Figure S10), the surface of LiAC‐500(10^−1^ m) is extensively covered with Li_2_CO_3_, whereas LiAC‐500(10^−4^ m) exhibits only a sparse distribution of Li_2_CO_3_, confirming that lowering the precursor concentration leads to an ultralow level of Li pre‐doping that is difficult to detect by conventional spectroscopic methods. Furthermore, depth profiling analysis provides insight into the coating thickness and spatial distribution of Li species. The region over which the LiCO_3_^−^ and CO_2_^−^ signal intensities decrease at the initial stage is broader for LiAC‐500(10^−1^ m), indicating the formation of a thicker Li‐containing layer compared to the LiAC‐500(10^−4^ m). Notably, the initial decrease of LiCO_3_^−^ and CO_2_^−^ signals near the surface suggests that Li species are predominantly localized at the surface rather than penetrating into the pore of AC. This result implies that even at higher precursor concentrations, Li compound formation is largely confined to the outer surface of AC, while reducing the precursor concentration enables the formation of an ultrathin and surface‐selective Li coating that preserves the intrinsic porous architecture.

### 2.3 Effect of Li‐Compound Types and Contents on Electrochemical Performance

**FIGURE 4:**
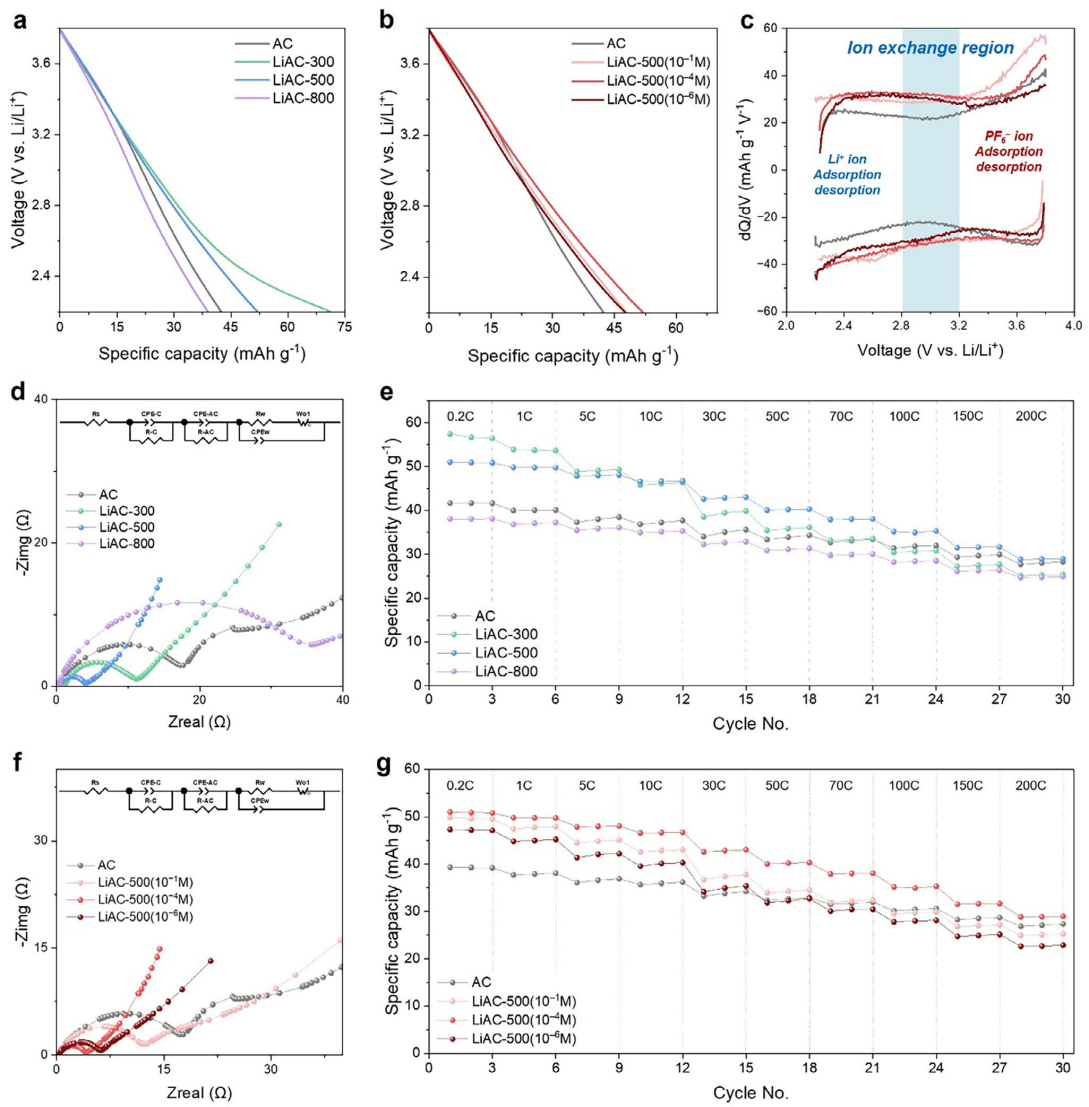
Electrochemical performance of the AC and LiACs in half‐cells. (a) Voltage profiles of AC, LiAC‐300, LiAC‐500, and LiAC‐800. (b) Voltage profiles and (c) corresponding dQ/dV curves of the AC and LiAC‐500 samples prepared with different precursor concentrations (10^−1^, 10^−4^, and 10^−6^ m). (d) Nyquist plots of AC, LiAC‐300, LiAC‐500, and LiAC‐800, with the equivalent circuit model shown in the inset. (e) Rate performance of the AC and LiACs at various C‐rates. (f) Nyquist plots and (g) rate capability of the AC and LiAC‐500s prepared at varying precursor concentrations.

The influence of heat‐treatment temperature and Li precursor concentration on the electrochemical performance of LiAC were investigated using a pouch type half‐cell configuration. As shown in Figure 4a, the voltage profiles of AC, LiAC‐300, LiAC‐500, and LiAC‐800 reveal a dependence of capacity on the thermal treatment conditions. Compared with pristine AC, the LiAC‐300 and LiAC‐500 exhibit noticeably enhanced capacities. In contrast, the LiAC‐800 shows a significant decrease in capacity, indicating that the formation of Li_2_O at elevated temperatures increases the interfacial resistance and suppresses the Li‐ion adsorption reaction below 3.2 V. Notably, the LiAC‐300 delivers the highest capacity among the investigated samples. This improvement is associated with the presence of ─COO^−^ functional groups generated during mild thermal treatment, which can facilitate the adsorption and storage of Li ions on the carbon surface. The influence of precursor concentration on the electrochemical behavior was also investigated. Since these samples were prepared with a heat‐treatment temperature of 500°C, all LiACs exhibit higher capacities than pristine AC, indicating the beneficial role of Li‐compound modification, while the LiAC‐500(10^−4^ m) shows the highest capacity among the samples. When the precursor concentration is relatively high, the deposited Li compounds partially block the pore structure of AC, thereby restricting Li‐ion adsorption. In contrast, when the precursor concentration is low, the Li‐compound coating becomes insufficient to uniformly cover the AC surface, leading to limited Li‐ion transport. As shown in the dQ/dV curves (Figure 4c), the Li‐doped samples exhibit intensified capacity below 3.0 V, a region associated with Li‐ion adsorption‐desorption reactions, indicating that Li pre‐doping facilitates Li‐ion transport. These results highlight that the chemical state and content of Li compounds determine Li‐ion transport and surface coverage within the AC, optimizing the Li‐ion storage capability of LiAC.

Electrochemical impedance spectroscopy (EIS) was conducted to elucidate the effect of Li doping on the interfacial charge‐transfer behavior (Figure 4d and Figure S11 and Table S1). The Nyquist plots show a progressive decrease in resistance upon Li incorporation, with LiAC‐500 exhibiting the smallest semicircle, indicative of the lowest charge‐transfer resistance. This improved kinetics is attributed to the formation of Li_2_CO_3_ at 500°C, which facilitates Li‐ion transport while preserving effective electronic conduction within the porous carbon matrix. Rate capability tests were performed over a wide range from 0.2C to 200C to evaluate the high‐power properties of the LiAC electrodes (Figure 4e). Although LiAC‐300 delivers the highest initial capacity, its rapid capacity decay at high rates indicates limited Li‐ion transport, suggesting that the –COOLi species primarily facilitate initial Li‐ion adsorption/desorption but do not provide efficient ion migration pathways. In contrast, LiAC‐500 exhibits superior rate performance, maintaining 57% of capacity retention at 200C, which is consistent with its lowest charge‐transfer resistance observed in EIS. Furthermore, the voltage profiles at different rates reveal noticeable distortion in the below 3.0 V region for LiAC‐300 and LiAC‐800 at high rates, whereas LiAC‐500 shows minimal change, indicating more facile Li‐ion transport under high‐rate conditions (Figure S12). This behavior can be attributed to the presence of Li_2_CO_3_, which effectively enhances Li‐ion transport while preserving electronic conductivity, thereby enabling robust high‐rate electrochemical performance. In addition, to exclude the influence of thermal treatment on the AC itself, pristine AC was heat‐treated at 500°C without Li loading (Figure S13). The heat‐treated AC (AC‐500) exhibited voltage profiles and resistance nearly identical to those of pristine AC, confirming that the enhanced performance of LiAC‐500 originates from the Li‐containing surface compounds rather than from thermal treatment alone.

In the Figure 4f, the Nyquist plot under different precursor concentration further confirms that LiAC‐500(10^−4^ m) exhibits the lowest charge‐transfer resistance, indicating the most favorable interfacial kinetics. This result suggests that both insufficient and excessive Li pre‐doping hinder Li‐ion transport, highlighting the existence of an optimal doping level. The rate capability tests also reveal that the LiAC‐500(10^−4^ m) sample delivers the highest capacity retention at 200C, demonstrating superior high‐rate performance (Figure 4g and Figure S14). To further refine the precursor concentration, additional LiAC‐500(10^−3^ m) and LiAC‐500(10^−5^ m) samples were evaluated (Figures S15 and S16). Although all three samples exhibited similar initial capacities and voltage profiles, the LiAC‐500(10^−4^ m) showed superior rate capability and interfacial kinetics, confirming it as the optimal concentration for uniform surface coverage without pore blockage. Additionally, to distinguish the effect of a small amount of Li_2_CO_3_ physically dispersed within the electrode, an electrode was prepared by mixing 2 wt.% Li_2_CO_3_ powder with pristine AC. The mixed electrode exhibited a voltage profile nearly identical to that of pristine AC and only a limited reduction in impedance, confirming that Li_2_CO_3_ facilitates Li‐ion transport only when it forms an interface directly with the AC surface (Figure S17). Consequently, the strong correlation between reduced interfacial resistance and enhanced rate capability underscores that optimized Li doping effectively facilitates Li‐ion transport while maintaining efficient electronic pathways within the electrode.

### 2.4 Molecular Dynamics Calculation of Li‐Ion Transport in Li‐containing Interfacial Phases

To further elucidate the origin of the enhanced interfacial kinetics observed for the Li‐doped AC electrodes, molecular dynamics simulations were performed using interfacial models in which amorphous Li_2_O or Li_2_CO_3_ was placed in direct contact with AC. The amorphous structure was adopted to emulate the trace‐level Li‐compound layers formed on the AC surface under the present Li pre‐doping conditions. These two Li‐containing phases were selected, in line with the experimental phase‐evolution. The suggested Li_2_CO_3_/AC and Li_2_O/AC models allow direct comparison of how the experimentally observed Li‐containing surface phases supply Li ions toward the AC pore environment. With representative structures, we performed MD calculations at 773K, which corresponds to the 500°C heat‐treatment condition used in forming the Li_2_CO_3_‐rich interfacial layer. Li‐ion hopping events are extracted from finite‐time trajectories and compared through mean‐squared displacement and Arrhenius plot analysis.

**FIGURE 5:**
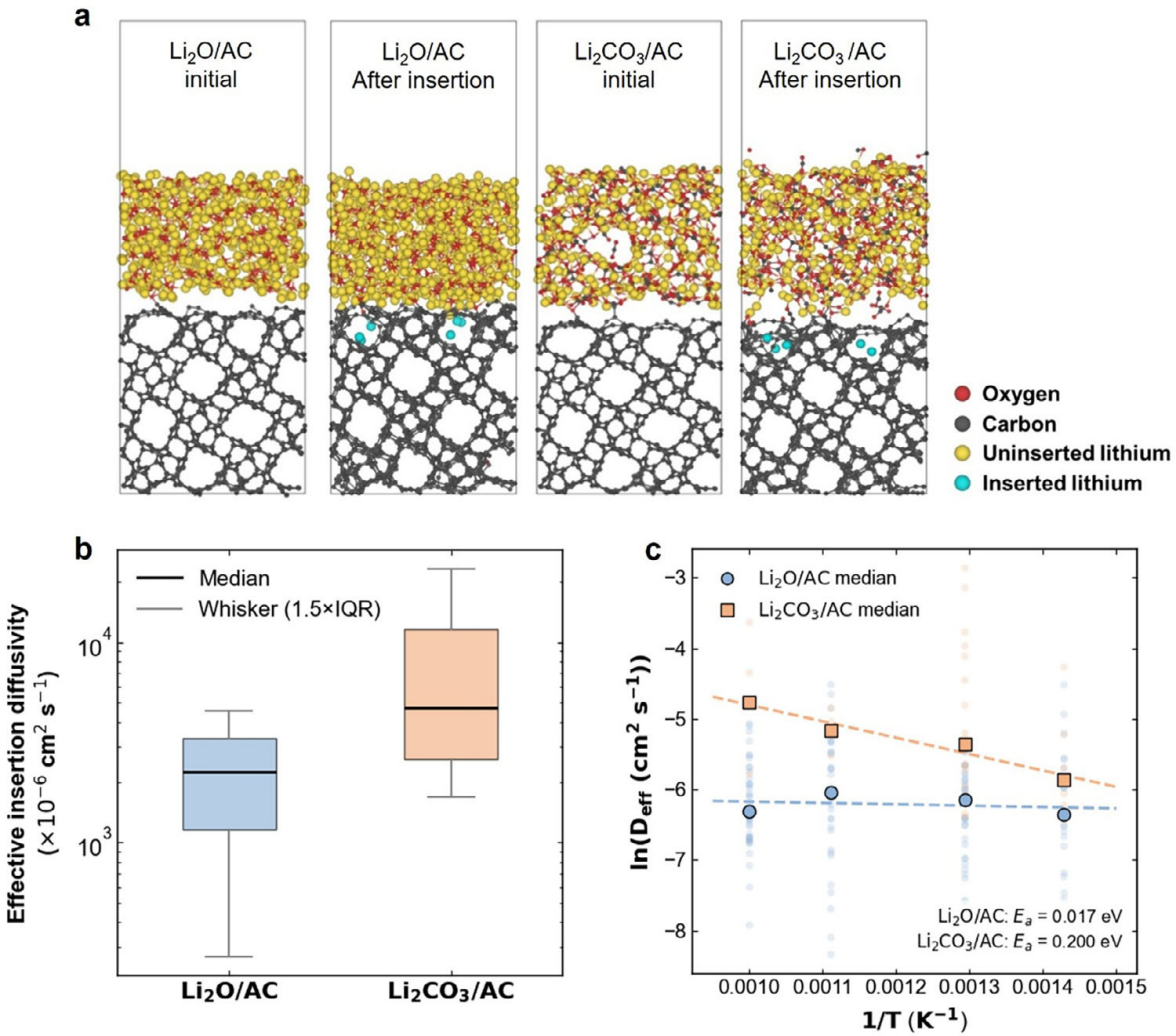
MD analysis of interfacial Li‐ion insertion from amorphous Li‐containing phases into activated carbon. (a) Initial and final configurations of the amorphous Li_2_O/AC and Li_2_CO_3_/AC interfacial model at 773 K. (b) Distributions of MSD‐derived effective insertion diffusivities (*D*_eff_), calculated using only the transient Li‐insertion segments into the AC region before the MSD reached a plateau. (c) Arrhenius plots of the effective insertion diffusivity for Li_2_O/AC and Li_2_CO_3_/AC models. The extracted values represent apparent interfacial insertion kinetics rather than bulk Li diffusion in isolated Li‐containing phases.

As shown in Figure 5a, the MD trajectories reveal Li‐ion migration from the amorphous Li‐containing phase toward the AC region. The Li displacement behavior could be divided into two distinct regimes: a rapid insertion regime, in which Li ions move from the Li‐containing phase into the carbon‐accessible pore region, followed by a plateau regime, in which the inserted Li ions become stabilized inside the AC void and undergo localized vibration. Because the experimentally relevant process is Li supply into the AC pore environment, the Mean‐Squared Displacement (MSD) analysis was performed only for the rapid insertion segment, while the plateau region associated with confined vibration was excluded from the fitting (Figure S18) [48, 49, 50]. The Li_2_CO_3_/AC interfacial model exhibited a higher MSD‐derived effective insertion diffusivity than the Li_2_O/AC model (Figure 5b), indicating that Li_2_CO_3_ more effectively promotes Li‐ion transfer into the carbon‐accessible region. This result is directly relevant to the electrochemical behavior of Li‐doped AC cathodes, because the improved high‐rate performance requires facile Li supply from the surface Li‐containing layer into the AC pore environment, rather than fast Li diffusion within the Li‐containing phase alone. Arrhenius analysis was further performed using the MSD‐derived effective insertion diffusivities obtained at different temperatures (Figure 5c). The extracted activation energy should therefore be interpreted as an apparent activation energy for interfacial Li insertion into the AC region, not as a bulk migration barrier within the isolated Li‐containing phase. Although the apparent activation energy for the Li_2_CO_3_/AC model was higher than that for the Li_2_O/AC model, the Li_2_CO_3_/AC interface showed higher effective insertion diffusivity over the investigated temperature range. This indicates that the overall Li insertion behavior is governed not only by the apparent energetic barrier, but also by the ability of the Li‐containing phase to release Li ions and provide connected migration pathways toward the AC pore region.

The enhanced Li insertion from Li_2_CO_3_ into AC can be attributed to the weaker Li─O Coulombic coupling in the carbonate‐based amorphous network. In Li_2_CO_3_, the negative charge of the CO_3_^2−^ anion is distributed over multiple oxygen atoms, reducing the effective charge density on each oxygen and weakening the Li─O interaction compared with oxide‐like Li_2_O. This weaker coordination allows Li ions to more readily detach from the Li_2_CO_3_ phase and migrate toward neighboring oxygen sites or the carbon‐accessible pore region. In contrast, the stronger Li─O interaction in Li_2_O can retain Li ions more strongly within the oxide‐like network, resulting in slower Li supply into AC. This interpretation is consistent with previous theoretical findings that amorphous Li_2_CO_3_ provides weaker Li─O coupling and more favorable Li‐ion transport characteristics than amorphous Li_2_O [51]. These simulation results explain why the optimized Li‐doped AC exhibits reduced charge‐transfer resistance and superior rate capability. Notably, the simulations suggest that the enhancement in Li‐ion diffusion originates from the formation of the Li_2_CO_3_/AC interfacial region itself, implying that the interfacial Li‐ion transport behavior remains effective once the interface is established, regardless of the overall Li‐compound loading. The Li_2_CO_3_‐rich interfacial domains formed under the optimized low‐precursor condition can supply Li ions more effectively into the AC pore environment, while their ultralow loading prevents pore blockage and preserves the conductive carbon framework. Thus, the performance enhancement of LiAC‐500 arises from an optimized interfacial chemistry that facilitates Li‐ion insertion into AC without sacrificing ion‐accessible porosity.

### 2.5 Full Cell Performance of LiAC Under Practical Conditions

**FIGURE 6:**
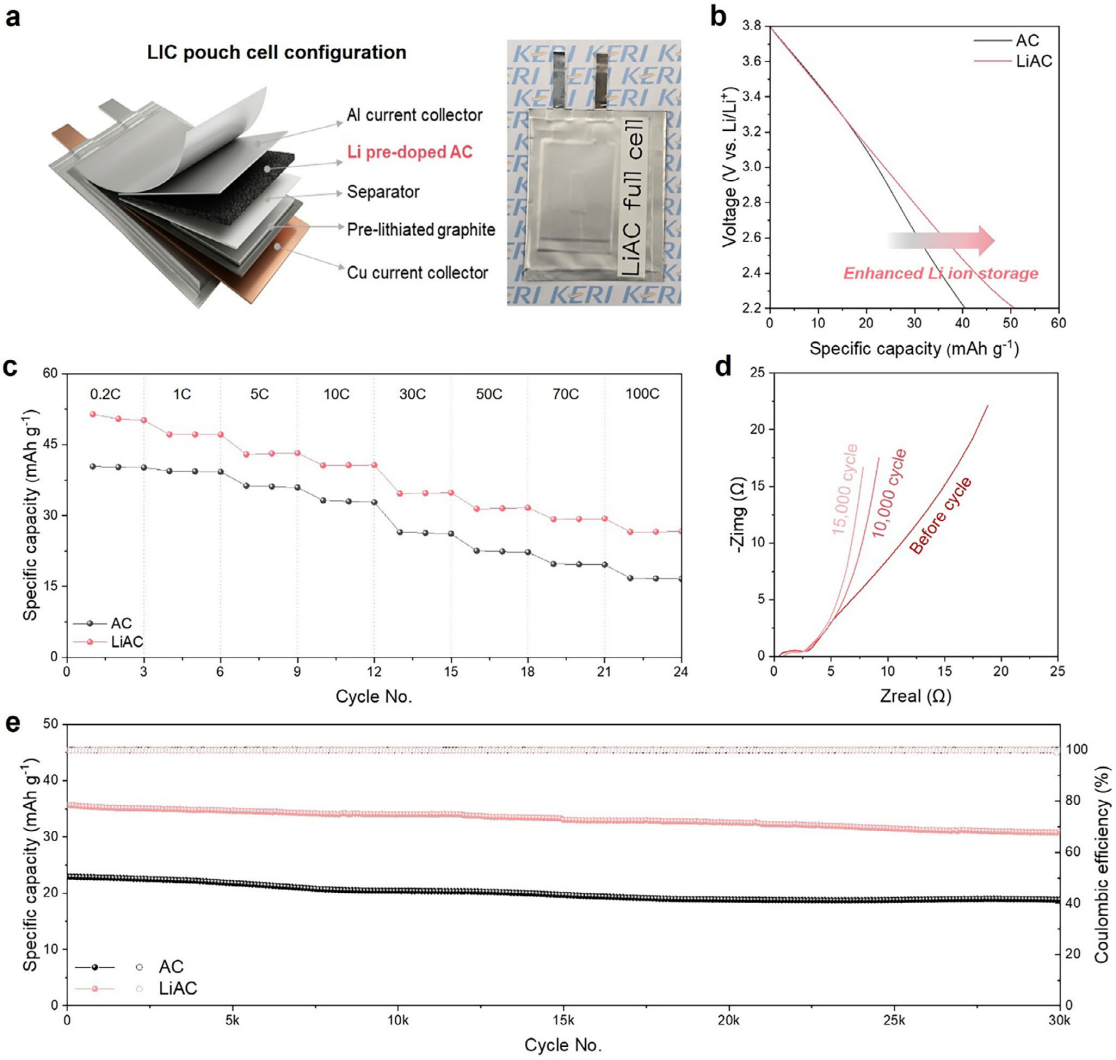
Practical pouch‐type LIC full‐cell performance. (a) Schematic illustration of the pouch‐type LIC full‐cell configuration and corresponding photograph of the fabricated pouch cell. (b) Voltage profiles of full cells assembled with pristine AC and LiAC cathodes. (c) Rate performance of AC and LiAC full cells at various current rates. (d) Nyquist plots of the LiAC full cell during cycling. (e) Cycling performance of AC and LiAC full cells at a practical condition of 30C.

To evaluate the performance of the optimized Li pre‐doped electrode under practical operating conditions, electrochemical tests were conducted using pouch‐type LIC full‐cell. Figure 6a illustrates the schematic configuration and corresponding photograph of the assembled LIC full cell, which was constructed using the optimized Li pre‐doped cathode, LiAC‐500(10^−4^ m), paired with a pre‐lithiated graphite anode to emulate practical operating conditions. As shown in Figure 6b, the voltage profiles of the full cells clearly demonstrate that the modified cathode delivers enhanced Li‐ion storage behavior, particularly in the low‐voltage region below 3.2 V. This is evidenced by the increased discharge capacity compared to the pristine AC, indicating that the pre‐doping strategy effectively facilitates Li‐ion transport and utilization even under full‐cell operating conditions. Furthermore, the rate capability of the full cells was evaluated over a wide range of current densities from 0.2C to 100C (Figure 6c). The LiAC cell consistently exhibits superior capacity retention under high‐rate conditions, highlighting its kinetic stability in high‐power applications. In the rate‐dependent voltage profiles (Figure S19), both cells show an increase in IR drop with increasing current density, as expected due to kinetic limitations. However, the LiAC cell maintains more stable and less distorted charge–discharge curves. This behavior indicates significantly improved Li‐ion accessibility and reduced polarization, which can be attributed to the optimized surface chemistry and enhanced ionic transport pathways introduced by the Li pre‐doping.

To further elucidate the kinetic evolution during cycling, EIS was conducted on the full cells at different cycles (Figure 6d). Notably, no significant increase in charge‐transfer resistance was observed even after prolonged cycles, despite the presence of the Li_2_CO_3_ coating layer, indicating that the Li_2_CO_3_ was applied as a trace‐level surface layer sufficiently thin to avoid additional interfacial resistance during repeated cycling. Furthermore, the Nyquist plots reveal a decline of the low‐frequency tail with increasing cycle number, indicating a gradual reduction in diffusion‐related polarization. This behavior suggests that the ionic transport within the electrode becomes efficient upon cycling. The impedance evolution is consistent with enhanced Li‐ion accessibility and the establishment of stabilized ion‐transport pathways in the LiAC electrode. Such kinetic evolution during cycling implies that the pre‐doped Li compounds not only contribute to initial charge compensation but also play a critical role in maintaining continuous and facile ion migration throughout prolonged operation. The cycling stability of the full cells was evaluated under a high‐rate condition of 30C to rigorously assess their performance in high‐power applications (Figure 6e and Figure S20). The LiAC cell delivered a specific capacity of 35 mAh g^−1^, which is notably higher than that of the pristine AC counterpart. Notably, the LiAC‐based cell exhibited exceptional cycling stability, maintaining a coulombic efficiency of 99.9% over more than 30 000 cycles with negligible capacity degradation. This outstanding long‐term stability under aggressive cycling conditions highlights the robustness of the Li pre‐doping strategy. Furthermore, the Ragone plot demonstrates that the LiAC‐based LIC delivers competitive energy and power densities compared with previously reported LIC systems (Figure S21). Collectively, these results demonstrate that Li pre‐doping effectively mitigates kinetic limitations by facilitating Li‐ion transport and suppressing diffusion‐induced polarization, thereby enabling both enhanced capacity and remarkable cycle stability even under high‐rate operation.

## 3 Conclusion

In this work, we present a facile and efficient strategy to enhance both the capacity and high‐rate capability of LICs via trace‐level Li pre‐doping on AC cathode. By employing Couette–Taylor mixing, Li precursors were uniformly adsorbed onto the AC surface, followed by controlled thermal decomposition to generate Li compounds. Notably, the Li_2_CO_3_ is identified as the most effective Li‐containing phase for facilitating Li‐ion transport. Subsequently, the amount of Li pre‐doping was precisely regulated by tuning the precursor concentration to an ultralow level, beyond the detection limit of conventional characterization techniques. Despite the minimal loading, MD simulations revealed that even an ultrathin Li_2_CO_3_ layer significantly facilitates Li‐ion transport across the electrode interface. This finding highlights the critical role of interfacial chemistry rather than bulk Li content in governing electrochemical performance. As a result, the optimized Li pre‐doping conditions enabled remarkable rate performance, delivering 57% capacity retention even at a high rate of 200C. Furthermore, to validate practical applicability, pouch‐type full cells were assembled using LiAC cathode and pre‐lithiated graphite anode, exhibiting remarkable cycling stability over 30 000 cycles at 30C. Overall, this study provides fundamental insights into the role of trace‐level Li pre‐doping in modulating interfacial ion transport and demonstrates a scalable approach to simultaneously improve power and energy densities. The proposed strategy offers a promising pathway for advancing next‐generation high‐power energy storage systems.

## 4 Experimental Section

### 4.1 Materials

AC (YP‐50F) was purchased from Kuraray. Polyvinylidene fluoride (PVdF, Solef S5130) was obtained from Solvay, and Super P conductive carbon was procured from TIMCAL. N‐methyl‐2‐pyrrolidone (NMP), and lithium hydroxide (LiOH) were purchased from Sigma–Aldrich. Natural graphite (AGP8) was purchased from BTR, and Carboxymethyl cellulose (CMC) from DKS Co. Ltd. Styrene–butadiene rubber (SBR, TRD‐102A) was obtained from JSR. Lithium metal foil was purchased from Honjo Metal Co., Japan), and polyethylene separator (WL16C) was procured from W‐Scope. The electrolyte consisted of 1 m LiPF_6_ in EC/EMC (3:7 = v/v) was purchased from Soulbrain Co., Ltd.

### 4.2 Synthesis of LiAC

AC (10 g) was first oxidized using an acid mixture composed of 60 mL of H_2_SO_4_ and 140 mL of HNO_3_. The suspension was stirred for 24 h to introduce oxygen functional groups on the carbon surface. After the oxidation process, the product was repeatedly washed with deionized water until the residual acid was removed and then vacuum‐dried at 100°C overnight. The obtained oxidized activated carbon was subsequently dispersed in a 10^−4^ m LiOH aqueous solution and stirred for 30 min. The suspension was then injected into a Couette–Taylor reactor, where the reactor was operated at 1400 rpm for 35 min with the chiller temperature maintained at 60°C. After the reaction, the product was washed several times with deionized water to remove residual LiOH. The particles were collected by centrifugation at 9000 rpm for 20 min at 5°C, and this washing–centrifugation process was repeated three times. The collected powder (LiAC) was subsequently vacuum‐dried at 100°C for 12 h. Finally, the dried powder was heat‐treated in a horizontal vacuum tube furnace under an Ar atmosphere (99.999%) with a heating rate of 2°C min^−1^ to the target temperatures (300°C, 500°C, or 800°C) and maintained for 2 h.

### 4.3 Characterization of Materials

The morphology of the samples was characterized by FE‐SEM (JEOL, JSM‐7619) and Cs‐corrected TEM (JEOL, ARM200F) to examine particle morphology, surface features, and local structural details. For TEM observation, the samples were ultrasonically dispersed in ethanol, drop‐cast onto a Cu grid, and subsequently dried in air. The elemental composition and its spatial distribution were analyzed by EF‐EELS (JEOL, ARM200F), and the surface chemical states were identified by XPS (Kratos Analytical Ltd., AXIS SUPRA+). Furthermore, surface composition and secondary ion fragments were investigated by TOF‐SIMS (IONTOF GmbH, M6) to obtain more detailed information on the surface species. TGA was performed under an argon atmosphere using an SDT 650 unit (TA Instruments) to assess thermal decomposition behavior and sample composition changes, with the temperature ramped from 100°C to 800°C at 2°C min^−1^.

### 4.4 Computational Details

Amorphous Li_2_O and Li_2_CO_3_ models were generated from the corresponding DFT‐optimized crystalline structures using a melt‐and‐quench molecular dynamics calculations. The optimized crystalline structures were expanded into 3 × 3 × 3 supercells, and the cell volumes were adjusted to give an initial density corresponding to 75% of the corresponding crystalline density, reflecting the lower density expected for amorphous phases. All density functional theory (DFT) calculations were carried out using the projector augmented wave (PAW) method as implemented in the Vienna ab initio Simulation Package (VASP) [52, 53]. The exchange–correlation interactions were described using the Perdew–Burke–Ernzerhof (PBE) functional within the generalized gradient approximation [54]. Structural optimizations of crystalline Li_2_O and Li_2_CO_3_ were performed with a plane‐wave kinetic energy cutoff of 600 eV. The electronic self‐consistency criterion was set to 10^−5^ eV, and ionic relaxations were continued until the residual forces on each atom were less than 0.01 eV/Å. Brillouin zone integrations were carried out using a Γ‐centered k‐point mesh with a spacing of 0.15 Å^−1^. MD calculations for amorphization were performed using the CHGNet machine‐learning interatomic potential under the NVT ensemble with a Berendsen thermostat [55, 56, 57]. Each system was rapidly heated from 300 to 4000 K over 4 ps, cooled to 1000 K over 1 ps, equilibrated at 1000 K for 7 ps, further cooled to 300 K over 1 ps, and finally equilibrated at 300 K for 7 ps. A time step of 1 fs was used throughout the calculations. The amorphous character of the resulting structures was confirmed by radial distribution function analysis. The disappearance of long‐range periodic peaks and convergence of the radial distribution function to unity at long distances indicated the loss of crystalline long‐range order. For amorphous Li_2_CO_3_, the local carbonate coordination was also examined to ensure that the generated amorphous model retained chemically meaningful CO_3_^−^ structural motifs.

### 4.5 Electrochemical Analysis

The cells were assembled in pouch‐type configuration. The cathode slurry was prepared by mixing active materials, Super P conductive carbon, and PVdF binder in a mass ratio of 85:8:7 using N‐methyl‐2‐pyrrolidone (NMP) as the solvent. The slurry was coated onto etched Al foil and vacuum dried at 120°C for 12 h. For the half cell, 500 µm of lithium metal was used for the anode. For the full cell, the anode slurry was prepared by mixing graphite, Super P conductive carbon, CMC and SBR in a mass ratio of 94:2:1.6:2.4 using deionized water as the solvent. The slurry was coated onto Cu foil and vacuum dried at 110°C for 12 h. Both electrodes were cut into 2.5 × 2.5 cm^2^. The LIC full cell was assembled in the following configuration: Al foil / AC cathode / separator / Li metal / graphite anode / Cu foil. The AC electrode served as the capacitive cathode, while the graphite electrode functioned as the battery‐type anode. The inserted Li metal layer acted as a Li source to enable the pre‐lithiation of the graphite anode, facilitating stable operation of the LIC system. A polyethylene (PE) separator was used, and the electrolyte consisted of 1.0 M LiPF_6_ in EC/EMC (3:7 by volume). The N/P ratio of the full cell was controlled to be approximately 9–10. Electrochemical measurements were performed using a battery cycler (Maccor, Series 4000) within a voltage window of 2.2–3.8 V vs. Li/Li^+^. For the formation process, the cells were cycled at 0.2 C for five charge–discharge cycles to stabilize the electrode interface. EIS was measured using ZIVE‐MP2 system with an amplitude of 10 mV over a frequency range of 5 mHz to 1 MHz after the formation cycles. Rate capability was evaluated under a fixed charge rate of 0.2 C, with discharge rates varied from 0.2 to 200 C for the half cells and 0.2 to 100 C for the full cells. The cycling stability of the full cells was evaluated at a charge/discharge rate of 30 C. The C‐rate was calculated based on the mass of the activated carbon cathode.

## Author Contributions

**Dong Gyun Im**: investigation. **Jihyeon Ryu**: methodology, formal analysis. **Seojin Jeon**: software. **Jaeik Hyun**: conceptualization, methodology, investigation, supervision, writing – review and editing. **Ki‐Hun Nam**: validation, investigation. **Byeong Guk Kim**: methodology, investigation. **Kyungmin Do**: writing – original draft, methodology, conceptualization, investigation, formal analysis. **Seung Yol Jeong**: investigation, validation. **Tae‐Hyun Kwon**: formal analysis. **Woosun Jang**: software, writing – review and editing. **Seong‐Yong Kim**: formal analysis. **Seungwook Eom**: writing – review and editing, supervision, conceptualization. **Sunhye Yang**: conceptualization, methodology, writing – review and editing, project administration, supervision. **Ick‐Jun Kim**: methodology, investigation. **Myoung Joon Hwang**: formal analysis.

## Conflicts of Interest

The authors declare no conflicts of interest.

## Supporting information




**Supporting File**: advs76990‐sup‐0001‐SuppMat.docx.

## Data Availability

The data that support the findings of this study are available from the corresponding author upon reasonable request.

## References

[1] S. Zhao , N. Khan , Y. Wen , and O. Trescases , “Server Power Management With Integrated Lithium‐Ion Ultracapacitor and Bi‐Directional DC‐DC Converter for Distributed UPS and Reactive Power Mitigation,” in IEEE Applied Power Electronics Conference and Exposition (IEEE, 2015), 960–965.

[2] S. Zhao , N. Khan , S. Nagarajan , and O. Trescases , “Lithium‐Ion‐Capacitor‐Based Distributed UPS Architecture for Reactive Power Mitigation and Phase Balancing in Datacenters,” IEEE Transactions on Power Electronics 34, no. 8 (2019): 7381–7396.

[3] S. Akbar , M. Rehan , L. Haiyang , I. Rafique , and H. Akbar , “A Brief Review on Graphene Applications in Rechargeable Lithium Ion Battery Electrode Materials,” Carbon Letters 28 (2018): 1–8.

[4] J. P. Pender , G. Jha , D. H. Youn , et al., “Electrode Degradation in Lithium‐Ion Batteries,” ACS Nano 14, no. 2 (2020): 1243–1295. 31895532 10.1021/acsnano.9b04365

[5] N. Nitta and G. Yushin , “High‐Capacity Anode Materials for Lithium‐Ion Batteries: Choice of Elements and Structures for Active Particles,” Particle & Particle Systems Characterization 31, no. 3 (2014): 317–336.

[6] N. Choudhary , C. Li , J. Moore , et al., “Asymmetric Supercapacitor Electrodes and Devices,” Advanced Materials 29, no. 21 (2017): 1605336. 10.1002/adma.201605336 28244158

[7] J. Chen and P. S. Lee , “Electrochemical Supercapacitors: From Mechanism Understanding to Multifunctional Applications,” Advanced Energy Materials 11, no. 6 (2021): 2003311.

[8] K. S. Poonam , A. Arora , and S. K. Tripathi , “Review of Supercapacitors: Materials and Devices,” Journal of Energy Storage 21 (2019): 801–825.

[9] P. Simon and Y. Gogotsi , “Materials for Electrochemical Capacitors,” Nature Materials 7, no. 11 (2008): 845–854. 18956000 10.1038/nmat2297

[10] D. Thillaikkarasi , S. Karthikeyan , R. Ramesh , P. Sengodan , D. Kavitha , and M. Muthubalasubramanian , “Electrochemical Performance of Various Activated Carbon‐Multi‐Walled Carbon Nanotubes Symmetric Supercapacitor Electrodes in Aqueous Electrolytes,” Carbon Letters 32, no. 6 (2022): 1481–1505.

[11] K. Naoi , S. Ishimoto , J. Miyamoto , and W. Naoi , “Second Generation ‘Nanohybrid Supercapacitor’: Evolution of Capacitive Energy Storage Devices,” Energy & Environmental Science 5, no. 11 (2012): 9363–9373.

[12] J. Liang and D.‐W. Wang , “Design Rationale and Device Configuration of Lithium‐Ion Capacitors,” Advanced Energy Materials 12, no. 25 (2022): 2200920.

[13] O. E. Eleri , F. Lou , and Z. Yu , “Lithium‐Ion Capacitors: A Review of Strategies Toward Enhancing the Performance of the Activated Carbon Cathode,” Batteries 9 (2023): 533.

[14] M. N. Rafat , Z. Otgonbayar , S.‐H. Yang , I.‐J. Kim , and W.‐C. Oh , “Effect of Extremely Short‐Sized MWCNT as Additive Material in High Surface Area Activated Carbon and Its Enhanced Electrical LIC Performance,” Molecules 27, no. 20 (2022): 7033. 36296623 10.3390/molecules27207033 PMC9607419

[15] J. Ryu , S. Yang , J. Back , S. Eom , and I.‐J. Kim , “Electrochemical Performances of a LiFePO_4_‐Based Heat‐Treated Activated Carbon Electrode,” Chemical Communications 58, no. 76 (2022): 10675–10678. 36063133 10.1039/d2cc01709a

[16] S. R. Sivakkumar and A. G. Pandolfo , “Evaluation of Lithium‐Ion Capacitors Assembled With Pre‐Lithiated Graphite Anode and Activated Carbon Cathode,” Electrochimica Acta 65 (2012): 280–287.

[17] L. Jin , C. Shen , A. Shellikeri , et al., “Progress and Perspectives on Pre‐Lithiation Technologies for Lithium Ion Capacitors,” Energy & Environmental Science 13, no. 8 (2020): 2341–2362.

[18] P. Zhu , D. Gastol , J. Marshall , R. Sommerville , V. Goodship , and E. Kendrick , “A Review of Current Collectors for Lithium‐Ion Batteries,” Journal of Power Sources 485 (2021): 229321.

[19] J. P. Zheng , “Energy Density Theory of Lithium‐Ion Capacitors,” Journal of The Electrochemical Society 168, no. 8 (2021): 080503.

[20] A. Parejo‐Tovar , C. Merlet , P. Ratajczak , and F. Béguin , “Operando Tracking of Ion Population Changes in the EDL Electrode of a Lithium‐Ion Capacitor During Its Charge/Discharge,” Energy Storage Materials 73 (2024): 103810.

[21] B.‐M. Lee , B.‐S. Choi , J.‐Y. Lee , S.‐K. Hong , J.‐S. Lee , and J.‐H. Choi , “Fabrication of Porous Carbon Beads From Polyacrylonitrile as Electrode Materials for Electric Double‐Layer Capacitors,” Carbon Letters 31, no. 1 (2021): 67–74.

[22] J. Zhu , Q. Ma , L. Kong , et al., “Graphitic Carbon Nitride Film Deposited With Nitrogen‐Doped Carbon Nanoparticles as Electrode for High‐Performance Supercapacitors,” Carbon Letters 34, no. 9 (2024): 2279–2290.

[23] R. Zhang , K. Zhang , Y. An , et al., “LiAlO_2_ Coated Activated Carbons via Liquid‐Phase Deposition and Sintering for High‐Voltage Lithium‐Ion Capacitors,” Materials Science and Engineering: B 317 (2025): 118247.

[24] Y. Gao , Z. Yang , Y. Wang , and X. Wang , “Boosting Capacitive Storage of Cathode for Lithium‐Ion Capacitors: Combining Pore Structure With P‐Doping,” Electrochimica Acta 368 (2021): 137646.

[25] D. Puthusseri , V. Aravindan , S. Madhavi , and S. Ogale , “Improving the Energy Density of Li‐Ion Capacitors Using Polymer‐Derived Porous Carbons as Cathode,” Electrochimica Acta 130 (2014): 766–770.

[26] O. E. Eleri , F. Huld , J. Pires , et al., “Revealing Mechanisms of Activated Carbon Capacity Fade in Lithium‐Ion Capacitors,” Electrochimica Acta 453 (2023): 142359.

[27] T. Saito , K. Kuwahara , S. Ishikawa , and S. Shiraishi , “Thermal Pore Stability of Activated Carbon Materials to Heat Treatment Above 1000°C and Lithium‐Ion Capacitors Using Heated Silicon‐Carbide‐Derived Carbon,” Electrochemistry 88, no. 2 (2020): 57–59.

[28] S. W. Seo , W. J. Ahn , Y.‐S. Lee , S. C. Kang , and J. S. Im , “Improvement of Capacitor Performance by Pitch‐Based Binder for a New Alternative to Polymer Binders,” Surfaces and Interfaces 37 (2023): 102726.

[29] J. L. Figueiredo , M. F. R. Pereira , M. M. A. Freitas , and J. J. M. Órfão , “Modification of the Surface Chemistry of Activated Carbons,” Carbon 37, no. 9 (1999): 1379–1389.

[30] B. K. Pradhan and N. K. Sandle , “Effect of Different Oxidizing Agent Treatments on the Surface Properties of Activated Carbons,” Carbon 37, no. 8 (1999): 1323–1332.

[31] Y. Otake and R. G. Jenkins , “Characterization of Oxygen‐Containing Surface Complexes Created on a Microporous Carbon by Air and Nitric Acid Treatment,” Carbon 31 (1993): 109–121.

[32] S. Yang , I.‐J. Kim , I.‐S. Choi , H. S. Kim , and Y. T. Kim , “Enhanced Specific Capacitance of Modified Needle Cokes by Controlling Oxidation Treatment,” Physica Scripta 139 (2010): 014019.

[33] T. Yoon , M. S. Milien , B. S. Parimalam , and B. L. Lucht , “Thermal Decomposition of the Solid Electrolyte Interphase (SEI) on Silicon Electrodes for Lithium Ion Batteries,” Chemistry of Materials 29, no. 7 (2017): 3237–3245.

[34] J.‐W. Kim and H.‐G. Lee , “Thermal and Carbothermic Decomposition of Na_2_CO_3_ and Li_2_CO_3_ ,” Metallurgical and Materials Transactions B 32, no. 1 (2001): 17–24.

[35] A. N. Timoshevskii , M. G. Ktalkherman , V. A. Emel'kin , B. A. Pozdnyakov , and A. P. Zamyatin , “High‐Temperature Decomposition of Lithium Carbonate at Atmospheric Pressure,” High Temperature 46, no. 3 (2008): 414–421.

[36] M. G. Ktalkherman , V. A. Emelkin , and B. A. Pozdnyakov , “Production of Lithium Oxide by Decompostion Lithium Carbonate in the Flow of a Heat Carrier,” Theoretical Foundations of Chemical Engineering 43, no. 1 (2009): 88–93.

[37] M. Schrimpf , J. Esteban , H. Warmeling , T. Färber , A. Behr , and A. J. Vorholt , “Taylor‐Couette Reactor: Principles, Design, and Applications,” AIChE Journal 67, no. 5 (2021): 17228.

[38] M. Seenivasan , C.‐C. Yang , S.‐H. Wu , et al., “Using a Couette–Taylor Vortex Flow Reactor to Prepare a Uniform and Highly Stable Li[Ni_0·80_Co_0·15_Al_0.05_]O_2_ Cathode Material,” Journal of Alloys and Compounds 857 (2021): 157594.

[39] A. Love , D. S. Lee , G. Gennari , et al., “A Continuous‐Flow Electrochemical Taylor Vortex Reactor: A Laboratory‐Scale High‐Throughput Flow Reactor With Enhanced Mixing for Scalable Electrosynthesis,” Organic Process Research & Development 25, no. 7 (2021): 1619–1627.

[40] K. N. Wood and G. Teeter , “XPS on Li‐Battery‐Related Compounds: Analysis of Inorganic SEI Phases and a Methodology for Charge Correction,” ACS Applied Energy Materials 1, no. 9 (2018): 4493–4504.

[41] R. Dedryvère , L. Gireaud , S. Grugeon , S. Laruelle , J.‐M. Tarascon , and D. Gonbeau , “Characterization of Lithium Alkyl Carbonates by X‐Ray Photoelectron Spectroscopy: Experimental and Theoretical Study,” The Journal of Physical Chemistry B 109, no. 33 (2005): 15868–15875. 16853016 10.1021/jp051626k

[42] T. I. T. Okpalugo , P. Papakonstantinou , H. Murphy , J. McLaughlin , and N. M. D. Brown , “High Resolution XPS Characterization of Chemical Functionalised MWCNTs and SWCNTs,” Carbon 43, no. 1 (2005): 153–161.

[43] A. Karen , K. Ito , and Y. Kubo , “TOF‐SIMS Analysis of Lithium Air Battery Discharge products Utilizing Gas Cluster Ion Beam Sputtering for Surface Stabilization,” Surface and Interface Analysis 46, no. S1 (2014): 344–347.

[44] F. Jia , X. Zhao , and Y. Zhao , “Advancements in ToF‐SIMS Imaging for Life Sciences,” Frontiers in Chemistry 11 (2023): 1237408. 37693171 10.3389/fchem.2023.1237408 PMC10483116

[45] Y. Zhao , S.‐K. Otto , T. Lombardo , et al., “Identification of Lithium Compounds on Surfaces of Lithium Metal Anode With Machine‐Learning‐Assisted Analysis of ToF‐SIMS Spectra,” ACS Applied Materials & Interfaces 15, no. 43 (2023): 50469–50478. 37852613 10.1021/acsami.3c09643 PMC10623505

[46] M. Mense , M. M. Bela , S. P. Kühn , et al., “ToF‐SIMS Sputter Depth Profiling of Interphases and Coatings on Lithium Metal Surfaces,” Communications Chemistry 8, no. 1 (2025): 31. 39900991 10.1038/s42004-025-01426-0 PMC11790834

[47] A. Karen , K. Ito , and Y. Kubo , “TOF‐SIMS Analysis of Lithium Reaction Products on Electrodes of Lithium Air Batteries,” e‐Journal of Surface Science and Nanotechnology 12 (2014): 75–78.

[48] J. Lowe and D. Siegel , “Modeling the Interface Between Lithium Metal and Its Native Oxide,” ACS applied materials & interfaces 12, no. 41 (2020): 46015–46026. 32929961 10.1021/acsami.0c12468

[49] S. Chae , H.‐K. Lim , and S. Lee , “Energy Landscapes for Lithium Incorporation and Diffusion in Multidomain Silicon Suboxide Anode Materials,” ACS Applied Materials & Interfaces 15 (2023): 57059–57069. 38015616 10.1021/acsami.3c12846

[50] X. He , Y. Zhu , A. Epstein , and Y. Mo , “Statistical Variances of Diffusional Properties From Ab Initio Molecular Dynamics Simulations,” npj Computational Materials 4, no. 1 (2018): 18.

[51] M. Jo , S. W. Jeong , and S. Lee , “Amorphous Lithium Carbonate as a Superior Lithium Transport Pathway in Inorganic Solid Electrolyte Interphases,” Journal of Alloys and Compounds 1039 (2025): 182922.

[52] G. Kresse and J. Furthmüller , “Efficient Iterative Schemes for Ab Initio Total‐energy Calculations Using a Plane‐wave Basis Set,” Physical review B 54, no. 16 (1996): 11169–11186. 10.1103/physrevb.54.11169 9984901

[53] P. Blöchl , “Projector Augmented‐Wave Method,” Physical review B 50 (1994): 17953. 10.1103/physrevb.50.17953 9976227

[54] J. Perdew , K. Burke , and M. Ernzerhof , “Generalized Gradient Approximation Made Simple,” Physical review letters 77, no. 18 (1996): 3865–3868. 10062328 10.1103/PhysRevLett.77.3865

[55] B. Deng , P. Zhong , K. Jun , et al., “CHGNet as a Pretrained Universal Neural Network Potential for Charge‐informed Atomistic Modelling,” Nature Machine Intelligence 5, no. 9 (2023): 1031–1041.

[56] A. Larsen , J. Mortensen , J. Blomqvist , et al., “The Atomic Simulation Environment—a Python Library for Working With Atoms,” Journal of Physics: Condensed Matter 29 (2017): 273002. 28323250 10.1088/1361-648X/aa680e

[57] H. Berendsen , J. Postma , W. Gunsteren , A. DiNola , and J. Haak , “Molecular Dynamics With Coupling to an External Bath,” The Journal of chemical physics 81, no. 8 (1984): 3684–3690.

