# Supplementary material for: Trace Li Pre‐Doping of Activated Carbon Cathode Enabling Accelerated Li‐Ion Transport for High‐Power Lithium‐Ion Capacitors
Source: Adv Sci (Weinh). 2026 Aug 10;13(59):e76990. doi: 10.1002/advs.76990 (PMC13455841; doi:10.1002/advs.76990)
Supplement: Supplementary file 1 [file ADVS-13-e76990-s001.docx]

**Supporting Information**

**Trace Li Pre-doping of Activated Carbon Cathode Enabling Accelerated Li-Ion Transport for High-Power Lithium-Ion Capacitors**

Kyungmin Do et al.

**Supplementary Note 1**.

Calculation of mean-squared displacements and statistical analysis

Molecular dynamics simulations were performed to evaluate interfacial Li-ion transport from the amorphous Li-containing phase into the activated carbon region. The initial interfacial model was constructed by placing the amorphous Li-containing phase in direct contact with the AC model, and the migration of Li ions from the Li-containing phase toward the carbon-accessible pore region was monitored during the MD trajectory.

Because the experimentally relevant process is Li supply from the surface Li-containing species into the AC pore environment, rather than bulk-like Li diffusion within the Li-containing phase, the MSD analysis was performed only for the trajectory segment corresponding to Li insertion into the AC region. In the MD trajectories, Li migration exhibited two distinct regimes: a rapid displacement regime during Li entry into the AC region, followed by a plateau regime after Li became stabilized inside the pore/void region and underwent localized vibration. Therefore, only the former insertion regime, where the MSD increased continuously before reaching the plateau, was used for diffusivity analysis, while the confined vibration regime was excluded. The mean-squared displacement of the selected Li ions was calculated as

$D= \frac{<MSD\left( \Delta t \right)>}{2d\Delta t}$,

where *d* is the dimensionality of the system (*d* = 3), and MSD(*Δt*) represents the time-dependent mean squared displacement of Li ions over a time interval *Δt*. The diffusion coefficient was extracted from the slope of the linear region of the MSD-time relationship. In particular, the MSD analysis was specifically performed for Li ions located within the porous carbon region to quantify Li transport from the amorphous Li-doped components into the porous carbon.

The temperature dependence of Li diffusivity was analyzed using the Arrhenius equation:

$D= D_{0}e^{-\frac{E_{a}}{k_{B}T}}$,

Where $D_{0}$, $E_{a}$, $k_{B}$, and $T$ denotes the pre-exponential factor, activation energy, Boltzmann constant, and temperature, respectively. Diffusion coefficients at each temperature were obtained from MSD analysis, and the activation energy for Li transport was estimated from linear fitting of the Arrhenius plot.

Considering the structural heterogeneity of the amorphous Li-containing phase/AC interface, multiple independent Li-ion insertion events were statistically analyzed to capture representative interfacial transport behavior. Because the calculated effective diffusivities can span a broad range depending on the local pore geometry and Li coordination environment, statistical analysis was performed on the logarithmic diffusivity distribution. Outliers were identified using the interquartile range criterion, and the median diffusivity of the filtered dataset was used as the representative value for Arrhenius analysis.

**Figure S1**. Surface SEM images of activated carbon-based samples with different surface treatments and heat-treatment temperatures: (a) pristine AC, (b) OxAC, and Li-modified AC after heat treatment at (c) 300 ºC, (d) 500 ºC, and (e) 800 ºC. Compared with pristine AC, OxAC shows a roughened surface morphology. After Li incorporation and subsequent heat treatment, the surface morphology evolves with more pronounced surface heterogeneity and particulate features appearing at higher temperatures.

**Figure S2.** TEM HAADF image and corresponding EELS elemental maps of carbon and lithium.

**Figure S3**. N_2_ adsorption-desorption isotherms of pristine AC and LiACs after heat treatment at different temperatures. LiAC-300 exhibits a reduced surface area due to surface coverage of the porous structure, whereas LiAC-500 and LiAC-800 show surface area comparable to pristine AC.

**Figure S4**. High-resolution (a) C 1s and (b) O 1s XPS spectra of pristine AC and OxAC with peak deconvolution. Compared with pristine AC, OxAC exhibits increased contributions in the C–O and O–C=O regions, indicating the introduction of oxygen-containing surface functionalities after oxidation. In particular, the increase in the O–C=O component is consistent with the formation of carboxyl-type functional groups, which are commonly reported to be generated during acid treatment of carbon materials.

**Figure S5**. ToF-SIMS chemical maps of LiAC samples heat-treated at different temperatures, showing the spatial distribution of characteristic Li-containing fragments: (a) C_2_H_3_O_2_^−^ for LiAC-300, (b) LiCO_3_^−^ for LiAC-500, and (c) LiO^−^ for LiAC-800. The dominant negative ion signals vary with heat-treatment temperature, indicating a temperature-dependent evolution of surface Li compounds from carboxylate-related species at 300 ºC to carbonate-related species at 500 ºC and oxide-related species at 800 ºC

**Figure S6.** Cross-sectional SEM images of the LiAC-500 sample at different magnifications.

**Figure S7**. (a) TEM image, (b,c) elemental maps, and (d) EELS spectra of the LiAC-500(10^−4^ M). No clear Li-related signal is detected in the present EELS analysis, indicating that the number of Li-containing species formed under this condition is substantially reduced. This result further suggests that any Li-containing surface layer, if formed, is extremely limited and likely too thin to be clearly resolved by EELS.

**Figure S8**. XPS spectrum of the sample prepared with a Li precursor concentration of 10^−4^ M. No discernible Li-related signal is observed within the detection range, indicating that Li-containing species are not present at a detectable level on the surface. This result is consistent with the EELS analysis, suggesting that the amount of Li-derived species is significantly reduced under the diluted precursor condition.

**Figure S9.** ^7^Li NMR spectrum of the LiAC-500 sample.

**Figure S10**. ToF-SIMS chemical maps of LiAC-500(10^−1^ M) and LiAC-500(10^−4^ M), showing the spatial distribution of (a,d) LiCO_3_^−^, (b,e) ^6^LiCO_3_^−^, and (c,f) LiCO_3_H^−^ fragments. Strong and broadly distributed signals are observed for the LiAC-500(10^−1^ M), whereas the corresponding signals are markedly weakened for the LiAC-500(10^−4^ M). Although no clear Li-related features were detected by XPS and EELS at LiAC-500(10^−4^ M), the weak ToF-SIMS signal indicate that trace amounts of Li-derived species still remain on the surface under the diluted precursor condition.

**Figure S11.** Fitted Nyquist plots corresponding to Figs. 4d and 4f.

**Table S1**. Fitted EIS parameters corresponding to the impedance spectra shown in Fig. 4d and f, using the equivalent circuit model.

**Figure S12**. Galvanostatic charge–discharge curves of (a) AC, (b) LiAC-300, (c) LiAC-500, and (d) LiAC-800 at various current rates (0.2C–200C), corresponding to Fig. 4e. The charge–discharge curves show systematic changes with increasing rate, reflecting the variation in voltage polarization under different current conditions.

**Figure S13.** (a) Voltage profiles of AC, AC-500, and LiAC-500. (b) Nyquist plots of AC, AC-500, and LiAC-500.

**Figure S14**. Galvanostatic charge–discharge curves of (a) AC, (b) LiAC-500(10^−1^ M), (c) LiAC-500(10^−4^ M), and (d) LiAC-500(10^−6^ M), at various current rates (0.2C–200C), corresponding to Fig. 4g. The separation between the charge and discharge curves varies with current rate, indicating rate-dependent polarization behavior.

**Figure S15.** Voltage profile of AC, LiAC-500(10^−3^ M), LiAC-500(10^−4^ M), and LiAC-500(10^−5^ M).

**Figure S16.** (a) Rate performance of the AC, LiAC-500(10^−3^ M), LiAC-500(10^−4^ M), and LiAC-500(10^−5^ M) at various C-rates. (b) DRT curves of LiAC-500(10^−3^ M), LiAC-500(10^−4^ M), and LiAC-500(10^−5^ M).

**Figure S17.** (a) Voltage profiles of AC, physically mixed AC + Li_2_CO_3_ electrode, and LiAC-500. (b) Nyquist plots of AC, physically mixed AC + Li_2_CO_3_ electrode, and LiAC-500.

**Figure S18**. Representative MSD profiles used for evaluating the effective interfacial Li-insertion diffusivity at 773 K. MSD trajectory of the amorphous (a) Li_2_O/AC and (b) Li_2_CO_3_/AC model. Color-shaded and grey-shaded region denotes the regime when rapid insertion occurs at the interface, and the regime when Li is trapped inside the pore and vibrating locally.

**Figure S19**. Charge­–discharge profiles of the full cells employing (a) AC and (b) LiAC-500(10^–4^M) cathodes at various current rates (0.2C–200C), corresponding to Fig. 6c. The voltage gap between charge and discharge changes with increasing current rate, consistent with different degrees of polarization at different current conditions.

**Figure S20**. Charge–discharge profiles of the full cells employing (a) AC and (b) LiAC-500(10^–4^ M) over selected cycles, corresponding to Fig. 6e. The voltage gap between charge and discharge remains largely unchanged with cycling, indicating stable polarization behavior over repeated cycles.

**Figure S21.** Ragone plot comparing the energy and power densities of this work with those of previously reported systems [S1–S7].

**Reference**

[S1] D. P. Dubal and P. Gómez-Romero, “All Nanocarbon Li-Ion Capacitor with High Energy and High Power Density,” Materials Today Energy 8, (2018): 109–117.

[S2] M. Granados-Moreno, G. Moreno-Fernández, R. Mysyk, and D. Carriazo, “A High-Energy Hybrid Lithium-Ion Capacitor Enabled by a Mixed Capacitive-Battery Storage LiFePO_4_–AC Cathode and a SnP_2_O_7_–rGO Anode,” Sustainable Energy & Fuels 7, (2023): 965–976.

[S3] T. Zhang, F. Zhang, L. Zhang, et al., “High Energy Density Li-Ion Capacitor Assembled with All Graphene-Based Electrodes,” Carbon 92, (2015): 106–118.

[S4] H. Zhang, X. Xu, H. Wang, et al., “Lithium Ion Capacitor with Identical Carbon Electrodes Yields 6 s Charging and 100 000 Cycles Stability with 1% Capacity Fade,” ACS Sustainable Chemistry & Engineering 7, (2019): 2867–2877.

[S5] Y.-H. Chou, W.-Y. Wu, J.-D. Wei, T.-W. Wu, and W.-S. Tseng, “Boosting Energy Storage Density of Lithium-Ion Hybrid Capacitors via 3D Graphene Nanoflake Integration,” RSC Advances 15, (2025): 38636–38645.

[S6] J. B. Cossio, J. L. Gómez-Urbano, S. Darlami-Magar, et al., “Valorisation of Bourbon Distillery Waste into Single-Source Hybrid Lithium-Ion Capacitors,” Materials Advances 7, (2026): 5384–5395.

[S7] L. Li, Z. Zhou, F. Xu, et al., “Purified Molybdenite Encapsulated in N-Doped Carbon Nanofibers as Binder-Free Anodes for Flexible Lithium-Ion Hybrid Capacitors,” Nanoscale 18, (2026): 8106–8114.
